# Retraction: Apple Peel Polyphenols and Their Beneficial Actions on Oxidative Stress and Inflammation

**DOI:** 10.1371/journal.pone.0267060

**Published:** 2022-04-14

**Authors:** The PLOS ONE Editors

Following the publication of this article [1], concerns were raised about results presented in Figs 4, 8, 9, and 10. Specifically, the following results appear more similar than would be expected from independent samples:

- The Fig 4D Occludin panel and the Fig 10A β-actin panel.
- Lanes 1–2 of the Fig 8A COX2 panel, lanes 1–2 of the Fig 8B COX2 panel, and lanes 1–2 of the Fig 8C COX2 panel.
- Lanes 3–4 of the Fig 8A COX2 panel and lanes 3–4 of the Fig 8B COX2 panel.
- The Fig 8A β-actin panel, the Fig 8B β-actin panel, the Fig 8C β-actin panel, and lanes 2–5 of the Fig 8D β-actin panel.
- Lanes 4–5 and lanes 6–7 of the Fig 9A NF-κB panel.
- Lanes 1–3 of the Fig 9A IκB panel and lanes 1–3 of the Fig 9B IκB panel.
- Lanes 1–5 of the Fig 9B NF-κB panel and lanes 1–5 of the Fig 9C NF-κB panel.
- Lanes 4–7 of the Fig 9B IκB panel and lanes 4–7 of the Fig 9C IκB panel.
- Lanes 2–3 and lanes 4–5 of the Fig 10A NRF2 panel.
- Lanes 1–5 and lanes 6–10 of the Fig 10C β-actin panel.

The corresponding author stated that the similarities between reference (housekeeping) protein blots are due to the stripping and re-probing of the same blot, and commented that some blot images were spliced to remove extra lanes and/or duplicate or triplicate samples from the panel.

The corresponding author provided image data to support the results presented in Figs 8A, 8B and 9A. The underlying data for Fig 8B confirm that the panel has been spliced, but the underlying data provided for Fig 8A and Fig 9A do not appear to match the published panels. Overall, the data did not resolve the concerns about these figures.

The corresponding author stated that the underlying data for other figures were provided to *PLOS ONE* in 2014 but are no longer available in the laboratory records. PLOS is unable to access the journal’s 2014 correspondence records for this case. We sincerely regret that this case was not resolved much sooner after the prior correspondence.

Although we have been unable to review the primary data for several figures of concern, the nature and extent of unresolved image data reporting concerns call into question the reliability of the article’s results and conclusions. Therefore, the *PLOS ONE* Editors retract this article.

EL did not agree with the retraction. MCD, AF, SD, AM, CG, YD, and ED either did not respond directly or could not be reached.
